# Sanguinaria Canadensis as a Therapeutic Agent

**Published:** 1849-01

**Authors:** J. L. Mothershead


THE NORTH-WESTERN

### MEDICAL AND SURGICAL JOURNAL.

Vol. I.] DECEMBER, 1848---JANUARY, 1849. [No. 5.

### Part I.—Original Communications.

### ARTICLE I.

*Sanguinaria Canadensis as a Therapeutic Agent.* An Article read before the Indianapolis Medical Society. By J. L. Mothershead, M. D.

The Sanguinaria Canadensis, well known also by the names of Blood-root and Puccon, grows abundantly throughout the United States, and is one of our earliest Spring flowers. It is, indeed, so generally known that a description of it is deemed superfluous: I shall, therefore, pass it over, and proceed at once to its medical properties.

Dr. Beach, speaking of Sanguinaria, says, "the root is emetic, cathartic, sudorific, and emmenagogue; detergent, expectorant, &c." Dr. Eberle says, "its powers have been variously represented, and as yet, do not appear well understood." Dr. Bigelow considers the root as an acrid narcotic. Dr. Bird says its medical properties are, in every respect, similar to those of the Peruvian bark. Dr. Barton valued it for its emetic and expectorant powers. Dr. Francis, of New York, speaks highly of it in protracted and distressing affections of the chest. Dr. Ives also speaks favorably of it in diseases of the lungs and liver; he also recommends it in influenza, whooping-cough, and croup. Dr. McBride, of South Carolina, recommends it in hydrothorax and asthmatic affections. Dr. Tully, speaking of the root, says, "it possesses deobstruent properties, without producing emesis or catharsis." Dr. Zollikoffer says, "it possesses active and useful properties, and that none can be considered superior to it in acute rheumatism," and reports the treatment of some half dozen cases to sustain his opinions. He gives for his authority in using it in acute rheumatism, Nathan Smith, M. D., and also speaks well of it as emetic, cathartic, diaphoretic, and expectorant. Professor Cox speaks of it as emetic and cathartic, but says, "it should be given with great caution." Dr. Downey says, "it is powerfully emetic in doses of fifteen or twenty grains, but that eight grains is a mild dose and but little inferior to ipicacuanha." He also remarks that the *Tinc.* is used to prevent intermitting fever, and a decoction of the root to cure the dysentery. In one case, he says, it acted powerfully upon the uterus, producing abortion, and suggests itsuse in amenorrhœa in females. The root has been used in gonorrhœa; also, for the bites of serpents, and in bilious diseases. Professor Wood, of Philadelphia, says, "it is an acrid emetic, with stimulant and narcotic powers. It has been given in typhoid pneumonia, catarrh, pertussis, croup, phthisis pulmonalis, rheumatism, jaundice, hydrothorax, and other affections." Dr. Pereira speaks of it in terms similar to those used by Professor Wood, but says, "it must be considered as a remedy of questionable propriety in jaundice." Dr. Buel, of our own State, uses it as an emetic, but does not consider it so acrid or dangerous as is generally believed. Professor Smith, of New Hampshire, states that he has cured "several cases of polypi of the soft kind by giving the powdered root as a snuff." It has been recommended by some other writers as a cure for ill-conditioned ulcers, tinea capitis, &c.

I have thus given, as briefly as possible, the various opinions of medical writers on Sanguinaria Canadensis; which seem to be as strange as they are various and contradictory. I have not done so for the purpose of entering into any discussion upon the views entertained, but merely to give a short sketch of its history as a medicinal agent.

The principal diseases in which I believe Sanguinaria appropriate, are of a chronic character; and the article, to have its full effect, should be used as an alterative. My experience in its use has been confined principally to chronic affections of the stomach, liver, and lungs; but I will make some remarks on its applicability to other diseases.

In the first place I will speak of it in connection with dyspepsia, jaundice and other chronic affections of the liver. In dyspepsia of aggravated and long standing, I have used it in the form of pills, from one to three grains, morning, noon and night: beginning with one grain and increasing gradually as the stomach will bear it. The stomach, in this way, will receive, in a few days, doses which at first would have been too nauseating to bear.

Dyspepsia, when application is made to the physician for relief, is generally of long standing, and usually the action of the liver involved to such an extent as to make it a complication of the liver and stomach; hence, to relieve the case, you must give remedies that will affect both. And here Sanguinaria comes to your relief in a peculiar way; for while it excites the action of the stomach, increasing its powers, removing flatulence, and many other distressing symptoms; the liver is at the same time being aroused from its lethargy; distressing constipation is gradually relieved, and, by a continuation of treatment, your patient restored.

It is proper here to remark, that the usual advice as to dieting, and many other directions to the dyspeptic, should not be overlooked,

I will here relate two cases of dyspepsia; both of long standing, and treatment accordingly. First: Mr. A., of Delphi, in this State, applied to me two years since, stating that he had entirely despaired of getting well; that he had had recourse to all the usual remedies for dyspepsia, and was well satisfied that he could not be cured. Upon examining his case, I found him with all the distressing symptoms of dyspepsia; liver torpid; much reduced in flesh, with a contracted yellow skin. After giving him a cathartic, the Sanguinaria was ordered three times per day. He was permitted to increase his diet, and in four weeks was much improved. A dispatch came for more medicine, which was furnished, and the course continued for some months, when he informed me he was well and could eat whatever he desired.

Some two or three months since, Mr. A. was in our city, and informed me that he was in the enjoyment of better health than he had ever enjoyed.

The second case was Mr. M., of Madison, in this State. He had suffered long with dyspepsia; had tried every thing, and done every thing, Homœpæthy not excepted, and as yet no relief. In this case a similar course to the above was pursued,and the result was equally flattering. He is now fleshy, in good health, and says that with a box of pills in his pocket he is safe.

In jaundice, it has acted almost as a specific, in my hands. After giving a portion or two of calomel or blue pill, I begin with the tincture of Sanguinaria, and the syrup of Sarsaparilla, combined in such proportions as to enable the patient to take from thirty to sixty drops of the tincture three times per day; and it is strange with what rapidity the yellow tinge leaves the eyes and skin; and in a few days the patient is entirely restored.

I could here report numerous cases, to sustain its use in jaundice, but will let one suffice.

Col. V., of Logansport, in this State, (many years since,) having suffered long with this disease; his physician having resorted to all the usual remedies, without any relief; being much reduced and enfeebled, with a dark yellow tinge of the eyes and surface; a yellowish brown skin, much contracted and shrivelled. In this condition, believing there was no relief, he despaired of recovery, and visited this place for the purpose of settling up his affairs. When, fortunately, he was induced to try the Sanguinaria as above directed, and in ten or twelve weeks was cured; when, to use his own language, he was "just snatched from the grave." I have found Sanguinaria, in a great variety of affections of the liver, when jaundice was not present, or the stomach complained of, but that ever variable state of the liver, with pain in its region, constipation of the bowels, and all the attendant symptoms of a want of its action; promptly relieve, and in many cases effect a complete cure. I will here state that the syrup of Sarsaparilla was added, more with a view of covering the unpleasant taste of the tincture, than for its remedial powers.

In diseases of the lungs, after the active stages of inflammation have passed by, leaving irritating and troublesome coughs, and in chronic affections of the lungs generally, I have found the Sanguinaria a valuable remedy; indeed, so potent did its powers appear, in a few cases of phthisis pulmonalis, that I almost fancied the disease no longer incurable. But, lo! the destroyer was at hand—disease usurped its power, and the consolation alone was left, that the sufferers had been greatly relieved and their days prolonged if only for a season. In affections of the lungs, it has been my practice to combine the tincture of Sanguinaria with the syrup of squills, paregoric, &c. In retention of, and suppressed catamenia, the Sanguinaria has also been a valuable remedy in my hands; in some cases, however, it disappointed my expectations, and I am not prepared to say, to what extent it can be relied upon in such cases.

I have also used the Sanguinaria, in tinea capitis, tetter, and many other affections of the skin with decided success. It may be applied either in the form of powder or a strong tincture to the affected part; but I prefer a mixture of the tincture and sweet oil in equal parts, with the addition of a few drops of kreosote.

It will be seen from the foregoing remarks, that in classing Sanguinaria, it has been my object to look upon it as an alterative. Let not that fact deter others from its use, for of all the articles in the Materia Medica, next to Mercury and its preparations, none in my opinion, can compare with it, in its powers to excite the action of the liver, and it has the advantage of the former in its capability of being used at all times, and continued without producing any of its unpleasant effects.

For the last ten years I have used Sanguinaria Canadensis in my practice, in various forms, and in a variety of diseases. I have done so with close attention, and whatsoever the result may hereafter prove in the hands of others, I am well satisfied of its virtues as a medicine, and as deserving a place in therapeutics, heretofore not awarded it.

